# Effect of Er:YAG, Co2 lasers, papain, and bromelain enzymes dentin treatment on shear bond strength of composite resin

**DOI:** 10.1002/cre2.651

**Published:** 2022-08-25

**Authors:** Farahnaz Sharafeddin, Sara Maroufi

**Affiliations:** ^1^ Department of Operative Dentistry, Biomaterials Research Center, School of Dentistry Shiraz University of Medical Sciences Shiraz Iran; ^2^ School of Dentistry Shiraz University of Medical Sciences Shiraz Iran

**Keywords:** bromelain, Co2 laser, erbium laser, papain, shear bond strength

## Abstract

**Objective:**

Effective bond strength of composite resin restoration leads to its durability, so evaluation of dentin surface treatment with enzymes and laser for higher bond strength is an important factor.

**Materials and Methods:**

Sixty human molar teeth were cut at a depth of 2 mm of occlusal part and divided into six groups (*n* = 10). G1: etched with 37% phosphoric, G2 and G3: 10% papain or bromelain enzymes were used on the dentinal surface, G4: 10% papain and bromelain enzyme mixture were used for. Then, the specimens were washed with distilled water. In G5 and G6: Er:YAG or Co2 lasers were used on the dentin surface. An adhesive system was applied and then nanohybrid composite was placed in teflon mold and light cured. Samples were subjected to a shear bond strength (SBS) test by universal testing machines. Statistical analysis was performed, using one‐way analysis of variance and Tukey HSD tests (*p* < .05).

**Results:**

The mean SBS in G1 was significantly higher in comparison with the other groups (*p* < .0001). On the other hand, a comparison of mean SBS between groups 2, 3, 4, and 5 shows no significant differences (*p* = .221). The mean SBS in group 6 (Co2 laser) was significantly lower in comparison with the other groups (*p* < .0001).

**Conclusion:**

Results showed that SBS of composite resin to dentin was not significantly affected, using either bromelain or papain 10% enzymes or erbium laser. Co2 laser had a negative effect on dentin and decreased the SBS. Phosphoric acid has the best result.

## 1 INTRODUCTION

Dentin treatment is an important factor in tooth color restorations (Sharafeddin et al., 2021). With the aim of eliminating the influence of organic matrix on the adhesion of composites to the dentin, various substances with the deproteinizing characteristic have been suggested (Lopes et al., 2007). The effects of deproteinization with bromelain and papain enzymes on the shear bond strength (SBS) have been evaluated (Chauhan et al., 2015; Maurer, 2001; Sharafeddin & Haghbin, 2019; Sharafeddin et al., 2021).

Papain is a natural cysteine protease enzyme, extracted from papaya, (Tadikonda et al., 2017; Tsuge & Nishimura, 1999) It has exhibited proteolytic function toward proteins, amino acid esters, short chain peptides, amide links, and is used in the fields of food and medicine (Amri & Mamboya, 2012). Bromelain is a proteolytic enzyme, extracted from pineapple. Proteases can catalyze the hydrolysis of proteins to amino acids. Bromelain can act like nonsteroidal anti‐inflammatory drugs, with fewer side effects. It is used for deep burns trauma and postsurgery wounds (Muhammad & Ahmad, 2017). It has been reported that the removal of collagen fiber of dentin and enamel with bromelain enzyme after acid etching can improve SBS (Chauhan et al., 2015).

The laser stimulates dentin by producing porosity, saving minerals, and preparing open tubules of dentin without forming a smear layer, and also sterilizes the surface (Davoudi et al., 2016).

The erbium laser can be used for cavity preparation and removing caries. This laser makes dentinal water evaporates and causes changes in collagen, which might in turn increase dentin permeability (Ferreira et al., 2010). In comparison with acid etching, laser treatment is less technique‐sensitive and has more control over the area that needs to be precisely etched (Davari et al., 2022). Previous studies reported that irregular dentine surfaces and dentine tubules were opened after Er:YAG laser irradiation, which increases the bonding area between the dentine and adhesive (Wang et al., 2022). The Er:YAG laser, in particular, can promote equal adhesion results to conventional bur treatments when using a self‐etching universal adhesive, when enamel and dentin cavities are prepared with optimized laser settings with regard to power, frequency, water amount. In this context, also finishing or rather smoothening the cavity surface with less powerful settings seems to be essential for optimal marginal adaptation and adhesion (Otero et al., 2021).

Studies showed that a Co2 laser can concentrate high levels of energy in a small area. Energy is then converted into heat and results in burning, melting, or vaporization of the respective area in dentin. Melting and recrystallization of dentin result in obstruction of dentinal tubules, thus dentin hypersensitivity might occur (Mozaffari et al., 2016; Rode et al., 2003).

It has been reported that 9.3 μm Co2 laser preparations of enamel, have adversely affected the adhesion of adhesives. This might be due to chemical changes in the substrate caused by the occurrence of high temperatures (∼1000°C) next to the melting of the surface, which may explain the smooth and glazed surface micromorphology after laser use. Contrarily, Er:YAG laser preparations do not exceed temperatures of 250−300°C and produce honeycomb patterns. Regarding dentin, both laser types delivered good results in terms of micromechanical adhesion in dentin without previous phosphoric acid etching (Otero et al., 2021).

High SBS is an important factor in the longevity of restorations, so the aim of this study was to evaluate the effects of papain and bromelain enzymes, erbium, and Co2 lasers on SBS of composite resin to dentin.

## 2 MATERIALS AND METHODS

In this in vitro study with an ethical approval code (IR.SUMS.DENTAL.REC.1398.037), 60 maxillary intact human molar teeth were stored in a thymol solution of 0.1% at 4°C for 1 month. Then, they were put in acrylic resin (Acropars; Marlic Medical Co.) at the level of cement enamel junction. Teeth were sectioned at a depth of 2 mm from dentino enamel junction at the occlusal part by the diamond disk. Dentin surface was homogenized using 600‐grit silicon carbide paper (Sharafeddin et al., 2021).

The 10% bromelain or papain enzyme solutions were prepared by mixing the enzyme powder (6 gr) with distilled water (60 ml) in the biochemistry lab at Shiraz University of Medical Sciences.

The teeth were divided into six groups (*n* = 10):

Group 1: The dentin surface was etched with 37% phosphoric acid for 15 s.

Group 2: The 10% papain enzyme was applied on the dentin surface for 60 s.

Group 3: The 10% bromelain enzyme was applied on the dentin surface for 60 s.

Group 4: The mixture of 10% papain and bromelain enzymes was applied for 60 s.

Group 5: The erbium laser (erbium‐doped yttrium aluminum garnet laser; Profile) with a setting of 10 Hz, 50 mj, 1.5 W, and distance of 20 mm with a circular tip was used on the dentin surface for 30 s (Saberi et al., 2018).

Group 6: The Co2 laser (US‐20D; Deka Dental Laser system) with a setting of 10 Hz, 80 mj, 1.5 W, and distance of 20 mm with a circular tip was used on the dentin surface for 30 s (Saberi et al., 2018). All the theet were washed with distilled water for 20s.

**Figure 1:**
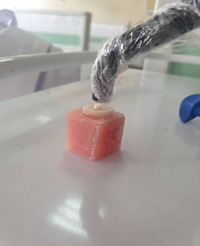
Curing step

**Figure 2:**
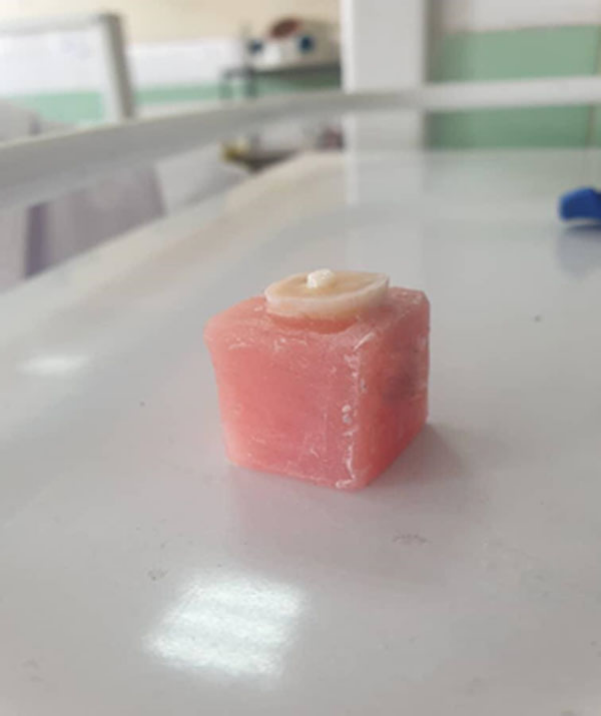
Composite restoration on the dentin surface

Adhesive systeme, Adper Single Bond 2 (3M ESPE, USA) was applied to the dentin surface in two layer and cured for 20 s. A nanohybrid composite (Z350 3M ESPE) was placed in 2 mm thick and 3 mm diameter teflon molds and light cured for 40s, using the light curing unit (Demi Plus; Kerr) with a light intensity of 1200 $\mathrm{mW}/{\mathrm{cm}}^{2}$ throughout the study. The distance between the composite resin and the light guide was less than 1 mm (Figures 1 and 2).

**Figure 3:**
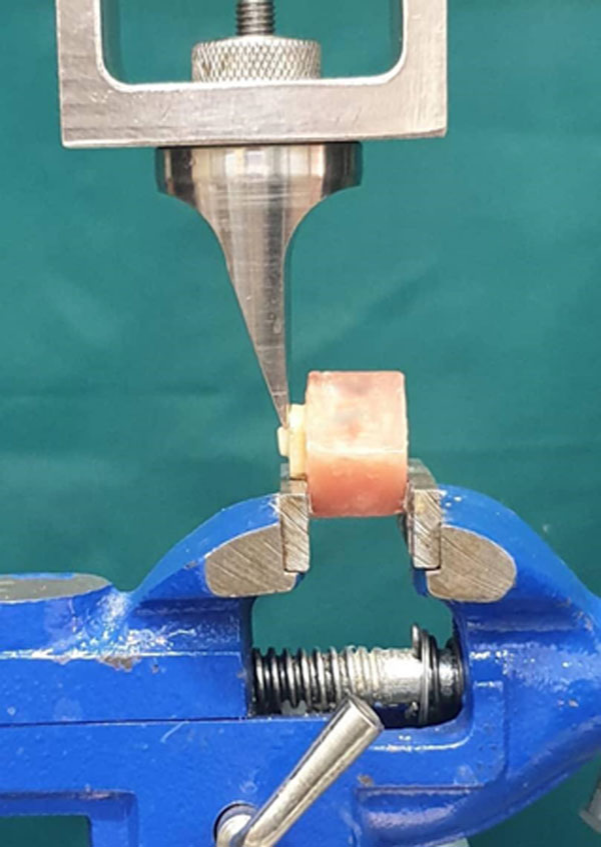
A sample in the universal testing machine

The specimens were stored in a 0.1% thymol solution for 24 h at room temperature. Then SBS test was done by a universal testing machine (Zwick/Roell Z020) at a crosshead speed of 1.0 mm/min (Figure 3). The force was recorded in Newtons and the SBS values were calculated from the peak load at failure divided by the specimen surface area in mega pascals (MPa).

The tested specimens were observed under a stereomicroscope (×40) (Motic K‐500L; Motic Incorporation Ltd) to evaluate the fracture mode. Fractures were evaluated as Type 1‐ adhesive: when fractured surface was between the composite and dentin; Type 2‐ cohesive: when fractured surface was in the composite block or dentin; and Type 3‐ adhesive/cohesive: when the fractured surface in the composite and dentin is considered a mix or adhesive/cohesive.

The statistical analysis was carried out using the statistical software (SPSS ver.22) and one‐way analysis of variance (ANOVA), post hoc, and Tukey HSD test (*p* < .05).

## 3 RESULTS

The descriptive statistics of SBS of etching or treatment of the dental surface, including mean, standard deviations, minimum, and maximum values in MPa are presented in Table 1.

**Table 1:** The shear bond strength (MPa) of study groups

Study groups	Mean ± SD	Minimum	Maximum
Group 1 Phosphoric acid 37%	13.35 ± 1.47	10.91	15.72
Group 2 Papain enzyme 10%	7.19 ± 1.22	5.54	9.75
Group 3 Bromelain enzyme 10%	7.62 ± 0.64	6.24	8.53
Group 4 Papain and bromelain enzyme 10%	8.04 ± 1.64	5.09	10.73
Group 5 Erbium laser	6.82 ± 0.90	5.34	8.33
Group 6 Co2 laser	4.44 ± 0.70	3.35	5.56

Abbreviations: MPa, mega pascals; SD, standard deviations.

**Figure 4:**
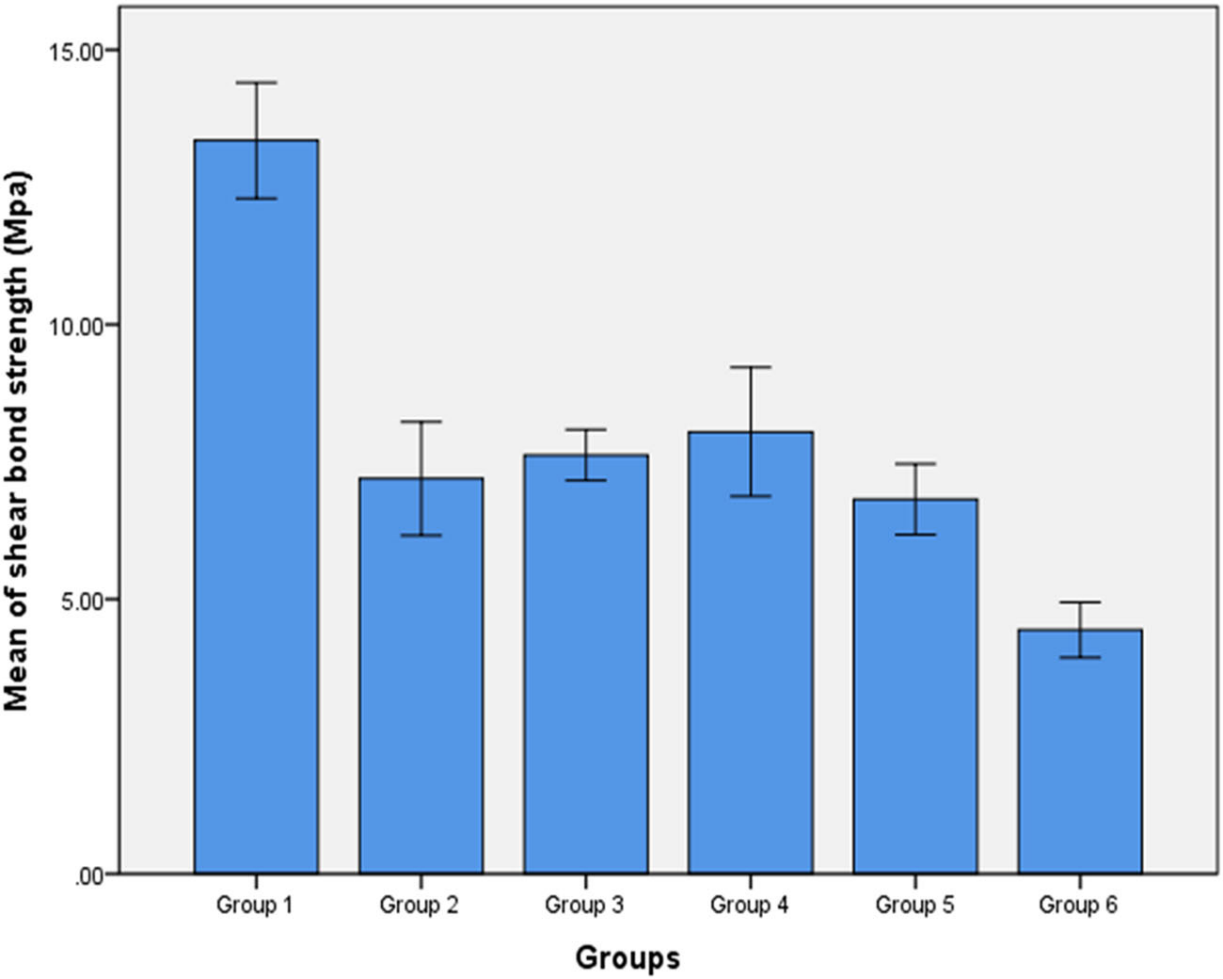
Means and standard deviations of the shear bond strength in the study groups. Group 1: Phosphoric acid 37%. Group 2: Papain enzyme 10%. Group 3: Bromelain enzyme 10%. Group 4: Papain and bromelain enzyme 10%. Group 5: Erbium laser. Group 6: Co2 laser.

One‐way ANOVA test was performed to compare the mean SBS values between the study groups (Figure 4). Our results indicate that the shear bond strength was significantly higher in group 1 (phosphoric acid 37%).

Comparing the mean shear SBS group 2 (papain enzyme 10%), group 3 (bromelain enzyme 10%), group 4 (papain and bromelain enzyme 10%), and group 5 (erbium laser) resulted in no significant differences (*p* = .221). In addition, the mean SBS in group 1 was significantly higher and obtained the best SBS result in comparison with the other groups (*p* < .0001). On the other hand, the mean SBS in group 6 (Co2 laser) was significantly lower in comparison with the other groups (*p* < .0001). For pairwise comparisons between the study groups, the Tukey HSD test was used (*p* < .05), (Table 2).

**Table 2:** Comparison *p* values of shear bond strength (Mpa) between the study groups

Study groups	Group 1	Group 2	Group 3	Group 4	Group 5	Group 6
Group 1	‐‐‐‐‐‐‐‐‐‐‐	0.0001	0.0001	0.0001	0.0001	0.0001
Group 2	0.0001	‐‐‐‐‐‐‐‐‐‐‐	0.966	0.614	0.982	0.0001
Group 3	0.0001	0.966	‐‐‐‐‐‐‐‐‐‐‐	0.969	0.669	0.0001
Group 4	0.0001	0.614	0.969	‐‐‐‐‐‐‐‐‐‐‐	0.221	0.0001
Group 5	0.0001	0.982	0.669	0.221	‐‐‐‐‐‐‐‐‐‐‐	0.0001
Group 6	0.0001	0.0001	0.0001	0.0001	0.001	‐‐‐‐‐‐‐‐‐‐‐

*Note*: Group 1: Phosphoric acid 37%. Group 2: Papain enzyme 10%. Group 3: Bromelain enzyme 10%. Group 4: Papain and bromelain enzyme 10%. Group 5: Erbium laser. Group 6: Co2 laser.

The fracture mode of the tested groups is shown in Table 3. Cohesive failure mode: Complete fracture of composite or tooth substrate. Adhesive failure mode: No signs of dentin fracture or remnants of resin on the tooth.

**Table 3:** Fracture mode of the study groups

Groups/mode of fracture	Groups	
	Cohesive	Adhesive
Group 1	8	2
Group 2	7	3
Group 3	6	4
Group 4	7	3
Group 5	5	5
Group 6	3	7

*Note*: Group 1: Phosphoric acid 37%. Group 2: Papain enzyme 10%. Group 3: Bromelain enzyme 10%. Group 4: Papain and bromelain enzyme 10%. Group 5: Erbium laser. Group 6: Co2 laser.

## 4 DISCUSSION

With the purpose to eliminate the influence of organic matrix on the adhesion of composites to the dentin, various substances with the deproteinizing characteristic have been suggested (Lopes et al., 2007). In recent years, there are several reports on determining the effects of deproteinization with bromelain and papain and enzymes on the SBS (Chauhan et al., 2015; Lopes et al., 2007).

In our study, a significant difference was observed in the SBS between the groups etched with 37% phosphoric acid in comparison to those etched with 10% papain enzyme. Several studies reported chemomechanical caries removal techniques like papain gel to have a negative effect on SBS of resin composite to dentin (Cecchin et al., 2010; Lopes et al., 2007). Also, it has been reported that the depth of dentin is an important factor in bond strength, and it decreases as the dentin depth increases (Bulut & Atsu, 2017). This might be related to the higher water content in deep dentin in comparison to superficial dentin, a result of the increasing diameters and numbers of dentinal tubules per unit area in deep dentin. Hence, we can conclude that the papain enzyme cannot significantly affect deep dentin.

The group etched with 37% phosphoric acid (group 1) has the highest SBS in comparison with the other groups. It has been reported that removal of unsupported collagen fiber and deproteinization of dentin surface with bromelain enzyme after acid etching has a positive effect on the SBS (Chauhan et al., 2015). In another study, the removal of collagen fiber with bromelain enzyme after acid etching has been evaluated and they found that it has a positive effect on SBS (Kochhar et al., 2018). All the mentioned studies showed that adding enzymes to the treatment of dentin surface after acid etching had an acceptable effect. On the contrary, in our study, the use of bromelain enzyme alone for removing unsupported collagen matrix was not as effective as phosphoric acid.

In another study, Sharafeddin & Safari (2019) reported that the papain enzyme on the dentin surface with the self‐etch adhesive system increased the SBS value. Also, bromelain application on dentin surface before two adhesive systems and papain before the total‐etch adhesive system had no effect on the SBS of composite to superficial dentin, which was in contrast to our findings. This difference shows that type of adhesive system has a greater effect on the SBS than the enzyme. In the abovementioned study, they used both etch‐and‐rise and self‐etch adhesive, but in our study, we used only etch‐and‐rinse adhesive.

We expected that the combination of bromelain and papain enzyme would enhance the SBS based on the reported study (Phiton et al., 2016). Although the SBS of the enzyme combination was higher than the other enzyme groups, this difference was not significant and it was lower in comparison to phosphoric acid. One reason for this could be that we excluded the effect of phosphoric acid on the deep dentin surface and merely focused on the effect of enzymes. Hence, we can conclude that although papain and bromelain enzymes had a collagenase effect, it was not comparable to phosphoric acid in removing inorganic tissues. Nonetheless, if they are used after etching dentin with phosphoric acid, it can increase the SBS.

Co2 and Er:YAG lasers are important and highly popular in dental treatment. Evidence shows that the smear layer is completely melted and evaporated following Er:YAG laser irradiation. On the other hand, the Co2 laser concentrates high levels of energy in a small area. It is then converted into heat that results in melting or burning, and vaporization of the affected area in the dentin. Melting and recrystallization of dentin results in obstruction of dentinal tubules and dentin hypersensitivity may be relieved (Mozaffari et al., 2016). It was reported that the highest bond strength results during the microtensile evaluation were found in laser followed by acid phosphoric usage. In these groups, the use of laser before the application of the adhesive resin was the preponderant factor for this increase in the bond strength (Oliveira et al., 2019). The difference in our study is due to the fact that we used laser irradiation alone for dentin treatment, while in their investigation, they used a laser followed by an acid etch to treat the dentin surface. The results of the present study showed that acid‐etched dentin surfaces can produce higher SBS than Er:YAG laser‐irradiated dentin surfaces, corroborating the findings of other studies (Eguro et al., 2002; Shahabi et al., 2013).

The Co2 laser group for dentin surface treatment showed lower SBS in comparison to the erbium laser group. It has been reported that, the morphological changes of dental tissue after Er:YAG, Co2 laser‐irradiation and phosphoric acid by means of scanning electron microscop evaluated and it was reported that Er:YAG laser can be an a good technique for surface preparation and can be act as safe as the conventional methods (Shahabi et al., 2013). However, Co2 laser has some adverse thermal side effects, making this device inappropriate for this purpose (Marracini et al., 2005). Therefore, it seems that the use of Co2 laser not only increases the SBS but can also have adverse effects. In general, in laser treatment, different factors might affect the SBS to tooth structure, such as the nature of the laser, wavelength, pulse duration, exposure time, laser power, amount of air steam created by water, and the distance from the laser tip to tooth structure (Shinoki et al., 2011).

The result of this study showed that the mean SBS in the erbium laser group was lower in comparison with enzyme groups, but this difference was not significant result, Juntavee et al. (2013) stated that microleakage was significantly higher, by Er:YAG laser in compare with the chemomechanical caries removal agent method, using Apacaries gel a preciousvaluable dental material which created of polyphenol in mangosteen extracts and spoon excavator and papain. It can be concluded that more microleakage leads to lower strength.

In the present study, cohesive failure occurred more in groups 1, 2, 3, 4, and 5, and adhesive failure occurred more in group 6. In line with our result, Perdigao et al. (2000) reported that in weaker adhesive agents, the failure type was adhesive, and the lowest resin penetration occurred in these types of adhesive systems. They also found that cohesive failure type was hapen with stronger adhesive systems. The idea that failure types were not correlated with bond strength values, especially in cohesive types of failures in dentin also has been reported by other researchers (Tezvergil et al., 2005).

This study is the first survey that evaluated the effects of bromelain, papain enzyme, Co2, and erbium laser on SBS of composite resin to dentin; therefore, it has several limitations. All specimens in this study were tested with one type of adhesive system and composite resin; therefore, it is possible that the other materials have different performances. There were limitations in the use of lasers for safety reasons and low intensities were used in our study. Further investigation with the large sample size, use of different concentrations of bromelain and papain enzyme, and different intensities of laser is required to develop the most appropriate method to increase the SBS of composite resin to dentin. Also, evaluation of the microtensile test is suggested for future research.

## 5 CONCLUSION

Within the limitation of the current study, it can be concluded that the SBS of 37% phosphoric acid was significantly higher and clinically acceptable. Although 10% papain and bromelain enzyme were effective in removing collagen matrix, it did not increase the SBS in comparison to 37% phosphoric acid. Also, the combination of enzymes did not have any influence on the SBS. The use of Co2 laser alone for dentin surface treatment incomposite restoration did not enhance the SBS and had a negative effect on it and decreased the SBS due to its thermal damage on dentin.

## AUTHOR CONTRIBUTIONS

Prof. Farahnaz Sharafeddin presented the conception and designed this research. Vice‐Chancellery of Shiraz University of Medical Science, Shiraz, Iran approved this research (No.19343) with the ethical approval code (IR.SUMS.DENTAL.REC.1398.037). Miss Sara Maroufi prepared the specimens and collected the data under the supervision of Prof. Farahnaz Sharafedin. Dr. Sayadi from Dental Research Development Center at Shiraz Dental Schools analyzed, and Prof. Farahnaz Sharafeddin and Miss Sara Maroufi interpreted the data. Prof. Farahnaz Sharafeddin and Miss Sara Maroufi drafted the article. Prof. Farahnaz Sharafeddin revised the article. Mr. H. Argasi from the Research Consultation Center of Shiraz University of Medical Sciences edited the article.

## CONFLICT OF INTEREST

The authors declare no conflict of interest.

## Data Availability

The data used to support the findings of this study are included in the manuscript.
